# Normal Values for Respiratory Oscillometry in Pediatrics: An Argument for a Local Control Population

**DOI:** 10.1002/ppul.71508

**Published:** 2026-02-27

**Authors:** Heather Boas, Joseph McDonough, Adam Lane, Sara B. DeMauro, Clement L. Ren, Maureen Josephson, Samuel B. Goldfarb, Paul D. Robinson, Julian L. Allen

**Affiliations:** ^1^ Division of Pulmonary and Sleep Medicine Children's Hospital of Philadelphia Philadelphia Pennsylvania USA; ^2^ Department of Pediatrics University of Pennsylvania Perelman School of Medicine Philadelphia Pennsylvania USA; ^3^ Division of Bone Marrow Transplantation and Immune Deficiency, Cancer and Blood Disease Institute Cincinnati Children's Hospital Medical Center Cincinnati Ohio USA; ^4^ Department of Pediatrics University of Cincinnati College of Medicine Cincinnati Ohio USA; ^5^ Department of Neonatology Children's Hospital of Philadelphia Philadelphia Pennsylvania USA; ^6^ Division of Pulmonary and Sleep Medicine, Department of Pediatrics University of Minnesota, Masonic Children's Hospital Minneapolis Minnesota USA; ^7^ Department of Respiratory Medicine Queensland Children's Hospital Brisbane Queensland Australia; ^8^ Children's Health and Environment Program, Child Health Research Centre University of Queensland Brisbane Queensland Australia

**Keywords:** predicted values, pulmonary function testing, reference equations, respiratory oscillometry, respiratory system reactance, respiratory system resistance

To the Editor,

Respiratory oscillometry is a test performed during normal tidal breathing, making it an ideal test of lung function for children too young to co‐ordinate the maneuvers required for spirometry [1]. The arrival of commercial devices has seen increasing interest in oscillometry as a test of respiratory system function; however, its integration into routine clinical care has been slow. There are several potential explanations for this reluctance to fully embrace oscillometry in clinical pulmonary function laboratories. Clinicians are more familiar with the spirometric and plethysmographic values of forced expiratory volume in 1 second, vital capacity, and resistance than certain parameters reflecting respiratory system high‐frequency oscillation mechanics, such as reactance or resonant frequency. The latter occurs at frequencies much higher than normal, or even abnormal, breathing frequencies, making its clinical relevance seem remote. In addition, the mathematics of oscillation mechanics can be daunting, as it relies on “complex numbers” with a real (resistive) and imaginary (reactive, encompassing both elastic and inertial properties) components. A recent publication attempts to present these concepts in a more intuitive way [2].

Another potential barrier to clinical implementation of oscillometry has been the lack of robust reference equations that generate normal predicted values accounting for growth from childhood to adulthood. How to best report results compared to normal, as percent predicted values versus *z*‐scores can also differ between pulmonary function laboratories. In addition, there are a number of different devices used in clinical care and research; these generate differing oscillatory signals, which may affect the results obtained.

The recent publication of equipment‐specific reference equations by Ducharme et al. for one such commercial device, the Tremoflo C‐100, which was derived from a large cohort of children aged 3–17 years of age at a single center in Montreal, Canada [3], represented an important step forward, as the first equipment‐specific pediatric equations for that device. However, the generalizability of these reference equations to other pediatric cohorts remains unclear. Generalizability of predicted equations to different local populations has been discussed extensively in the context of the Global Lung Function (GLI) initiative for spirometry [4]. We therefore explored the applicability of the Montreal reference equations to a local healthy control population, aged 4–18 years, tested at the Children's Hospital of Philadelphia in Philadelphia, USA.

Oscillometry was performed on a cohort of 80 healthy subjects using the same model Tremoflo device as was used in the Ducharme study. Approval was obtained by the Children's Hospital of Philadelphia Institutional Review Board (IRB 20‐018357_PERC). Parents or guardians provided written informed consent, and children aged 8 years and older provided assent for study participation.

Airwave oscillometry was performed using the Tremoflo C‐100 (Thorasys Thoracic Medical Systems Inc., Montreal, QC, Canada), according to European Respiratory Society (ERS) guidelines [5]. Before testing each subject, a device calibration check was performed according to the manufacturer's guidelines. During testing, subjects sat upright and comfortably, wearing nose clips, with their chins in “sniffing” position and with their cheeks supported. A minimum of three to five 30‐second trials were performed until at least three trials were obtained with a visually observed and recorded regular respiratory pattern, with no evidence of artifact (air leak, mouthpiece occlusion by tongue, coughing, or other glottic closure). Test results were reviewed to ensure satisfaction of ERS guidelines with a coefficient of variation < 10% in children over 10 years old, and < 15% in children 10 years and younger [5]. Primary oscillometry outcomes were resistance and reactance at 5 and 11 Hz (R5, R11, X5, and X11, respectively), resonant frequency (Fres), and the area under the reactance curve (AX).

Children without a history of prematurity, chronic medical conditions, cardiopulmonary history, or recent viral or respiratory illness within 4 weeks prior to the study were recruited. The mean age was 9.8 years, and 38 subjects (47.5%) were female. Additional characteristics and demographics are included in Table 1 alongside reported characteristics of Ducharme's Montreal population.

**Table 1:** Demographics of Philadelphia and Montreal subjects.

Characteristic	Philadelphia population (*n* = 80)	Montreal population (*n* = 306)
Age (years), mean (SD)	9.76 (3.62)	9.95 (3.61)
Male, *n* (%)	42 (52.5)	164 (53.6)
Height (cm), mean (SD)	140 (18.8)	142 (21.8)
Weight (kg), mean (SD)	39.9 (18.2)	37.4 (15.5)
BMI, mean (SD)	19.4 (5.01)	17.6 (2.72)
*Race and Ethnicity, n (%)* a		
Asian	10 (12.5)	
Black	17 (21.3)	25 (8.2)
White	56 (70)	212 (69.3)
Not Reported	1 (1.3)	
Hispanic or Latino	5 (6.3)	
Other^b^		69 (22.5)

^a^ Race and ethnicity were recorded differently between the two cohorts. Philadelphia totals are greater than *n* = 80 and 100% due to some subjects self‐identifying as multiple categories.

^b^ Additional details describing the “Other” category as defined by Ducharme et al. were not available.

One sample, 2‐tailed *t*‐tests were performed to determine if the means of the Philadelphia individual *z*‐scores for each measured outcome compared to the Ducharme predicted were significantly different than zero. Representative results for *z*‐scores of respiratory system resistance and reactance at 5 Hz compared to Ducharme are presented in the Figure 1. The mean *z*‐scores of the Philadelphia population calculated from the Montreal population data (R5 = −0.96, X5 = 0.67) were significantly different than zero (*p* < 0.0001) for both resistance and reactance, as well as Fres and AX (Table 2).

**Figure 1:**
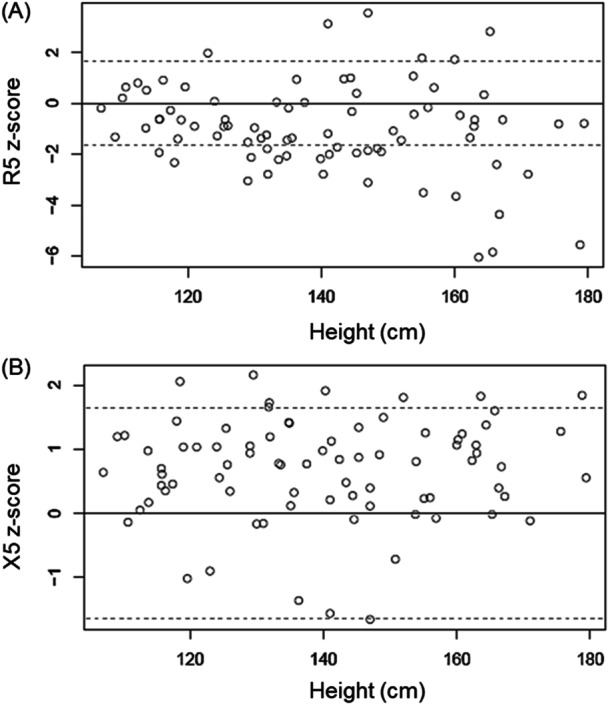
Distribution of Philadelphia dataset (open circles) as compared to *z*‐scores for R5 (A) and X5 (B) using the Montreal equations. The upper and lower dotted lines represent *z*‐scores of 1.65 and −1.65, respectively.

**Table 2:** Oscillometry *Z*‐scores, Philadelphia (*N* = 80), compared to Montreal.

Outcome	Mean *Z*‐score	SD	*p* value
R5	(−) 0.96	1.78	< 0.001
R11	(−) 0.51	2.04	0.028
X5	(+) 0.67	0.79	< 0.001
X11	(+) 0.74	0.83	< 0.001
AX	(−) 0.68	1.08	< 0.001
Fres	(−) 0.50	1.32	0.001

Although the mean *z*‐score differences between the two groups are all less than one, they still might represent a clinically significant center effect between Montreal and Philadelphia.

*Z*‐score shifts of less than one can impact the assessment of future risk. There have been a number of spirometry studies describing different risk trajectories in lung function in children and the development of later chronic obstructive pulmonary disease (COPD), depending on childhood *z*‐score. Bui et al. demonstrated a population of children with “normal” lung function with a *z*‐score between 0 and −0.5 at age 7 years, who had an accelerated decline in their lung function starting at approximately age 17 years [6]. Therefore, an overall *z*‐score shift of less than one in the Philadelphia population could represent clinically significant implications for estimations of future impairment.

The reasons for the center effect seen in oscillometric outcomes between children in Philadelphia compared to those in Montreal are unclear. The instruments used to perform the measurements were the same model made by the same manufacturer. Selection bias or sampling error may have played a role. But similar to Montreal, we used rigorous questionnaires to screen for prematurity and underlying cardiopulmonary disease. There was only a mean 0.2‐year difference in age and a mean 2 cm difference in height between the Montreal and Philadelphia cohorts (Table 1); however, since *z*‐scores are adjusted for height, even the small differences in height between the two centers are unlikely to provide an explanation for the differing *z*‐scores. Ducharme's screening criteria eliminated children who were obese (BMI > 97%ile) from their study, while ours did not; our Philadelphia cohort did have a higher mean weight and BMI and wider ranges as compared to the Montreal cohort (Table 1), and 12 subjects (15%) had BMI > 97%ile. However, we think that if anything, this should have caused higher resistance and more negative reactance in the Philadelphia cohort; we found the opposite. Race and ethnicity differences between the two cities could provide a possible explanation. However, while height was shown to be a significant predictor in Ducharme's oscillometry predicted equations, race and ethnicity were not. Socioeconomic factors and environmental exposures are also likely different between the two groups, which could lead to changes in lung function even in individuals considered healthy. Finally, there may be possible between device differences in Tremoflo C100 equipment.

A limitation of the present work is that it is a relatively small sample from a single institution. The presence of a center effect, if confirmed by further investigations, would indicate that a multicenter approach might be required to develop accurate reference equations.

In addition, these findings point to the importance, when feasible, of centers comparing their oscillometry results to a local control population, ideally tested at the same center, to determine the applicability of published reference values to the local population. The differences noted between healthy cohorts reinforce the value of current efforts to collate international datasets to produce robust, generalizable GLI reference data for oscillometry.

## Author Contributions

**Heather Boas:** conceptualization, investigation, writing − original draft, writing − review and editing, funding acquisition, methodology, formal analysis. **Joseph McDonough:** investigation, resources, writing − review and editing. **Adam Lane:** formal analysis, writing − review and editing, visualization. **Sara B. DeMauro:** resources, writing − review and editing, funding acquisition. **Clement L. Ren:** investigation, writing − review and editing. **Maureen Josephson:** writing − review and editing. **Samuel B. Goldfarb:** conceptualization, writing − review and editing, funding acquisition, resources, methodology. **Paul D. Robinson:** conceptualization, writing − review and editing, resources, methodology. **Julian L. Allen:** conceptualization, methodology, investigation, writing − original draft, writing − review and editing, funding acquisition, formal analysis, supervision, project administration.

## Conflicts of Interest

The authors declare no conflicts of interest.

## Data Availability

The authors have nothing to report.
